# Development and validation of a hypoxia-associated signature for lung adenocarcinoma

**DOI:** 10.1038/s41598-022-05385-7

**Published:** 2022-01-25

**Authors:** Brian Lane, Mairah T. Khan, Ananya Choudhury, Ahmed Salem, Catharine M. L. West

**Affiliations:** 1 Translational Radiobiology Group, Division of Cancer Sciences, University of Manchester, Manchester Academic Health Science Centre, Christie NHS Foundation Trust Hospital, Manchester, M20 4BX UK; 2 Department Clinical Oncology, Christie NHS Foundation Trust Hospital, Manchester, M204BX UK

**Keywords:** Cancer, Computational biology and bioinformatics, Biomarkers, Oncology

## Abstract

Hypoxia is common in non-small cell lung cancer (NSCLC) and an attractive therapeutic target. As hypoxia-targeting treatments are effective in patients with the most hypoxic tumours, we aimed to develop a lung adenocarcinoma (LUAD) hypoxia-related gene expression signature. RNAseq was used to identify genes significantly differentially expressed under hypoxia (1% O_2_) in four LUAD cell lines. Identified genes were used for unsupervised clustering of a TCGA-LUAD training dataset (n = 252) and in a machine learning approach to build a hypoxia-related signature. Thirty-five genes were upregulated in common in three of the four lines and reduced in the training cohort to a 28-gene signature. The signature was prognostic in the TCGA training (HR 2.12, 95% CI 1.34–3.37, p = 0.0011) and test (n = 250; HR 2.13, 95% CI 1.32–3.45, p = 0.0016) datasets. The signature was prognostic for overall survival in a meta-analysis of nine other datasets (n = 1257; HR 2.08, 95% CI 1.60–2.70, p < 0.0001). The 28-gene LUAD hypoxia related signature can be taken forward for further validation using a suitable gene expression platform.

## Introduction

Lung cancer is the most common cause of cancer-related death with approximately 1.8 million deaths worldwide in 2020^1^. Non-small cell lung cancer (NSCLC) accounts for 80–85% of all lung cancers, with the two main subtypes being adenocarcinoma (LUAD) and squamous cell carcinoma (LUSC)^2,3^. Positron emission tomography and oxygen electrode studies showed that hypoxia is widespread in NSCLC, which makes it an attractive therapeutic target^4^. However, clinical trials combining hypoxia-modifying therapy with radiotherapy did not show significant survival benefit^4,5^. This failure is in part due to hypoxia-modifying therapies being given regardless of the hypoxia status of the tumour^4,5^. Subgroup analyses of clinical trial cohorts measured hypoxia retrospectively using gene signatures^5,6^ or prospectively using other approaches^6^. These analyses showed hypoxia-modifying therapies only benefited patients with the most hypoxic tumours.

Gene signatures have been developed to assess hypoxia for a number of cancers, and some were extensively validated^6,7^. However, the derived LUAD hypoxia signatures were either not developed specifically for LUAD^8^, validated in one dataset^9^ or generated specifically for early stage LUAD^10,11^. A 51-gene hypoxia-associated signature developed for head and neck and breast cancer was prognostic in NSCLC datasets^8^. A potentially promising 4-gene hypoxia-related LUAD signature was derived using a list of published hypoxia-associated genes and then training on prognosis, but tested and validated in only one independent dataset^9^. A 16-gene mRNA hypoxia-related LUAD signature was developed for stage 1 and 2 LUAD patients using a gene co-expression network approach to identify genes associated with hallmark hypoxia gene enrichment scores and prognosis^10^. A 10-gene hypoxia-associated signature was then developed for stage 1 LUAD using co-expression networks to identify genes intersecting hypoxia-associated modules and HIF-1α targets followed by training on prognosis^11^.

As we previously successfully developed hypoxia-associated signatures for other cancers using genes identified as hypoxia-inducible in cell lines^12,13^, we aimed to use the approach for LUAD and LUSC and evaluate whether it could build on and improve previous signatures in terms of validating in multiple independent datasets.

## Results

### Induction of genes under hypoxia in LUAD cell lines

RNA-seq data were generated for four LUAD cell lines (A549, NCI-H2122, NCI-H1838, NCI-H1395) in biological triplicate under 1% O_2_ and 21% O_2._ Based on principal component analyses, one hypoxia sample for the NCI-H1395 cell line was identified as an outlier and removed (Supplementary Fig. 1). There were 205, 35 and 6 protein-coding genes up-regulated across at least two, three or all four LUAD cell lines, respectively (FDR < 0.05). There were no down-regulated genes in common across the cell lines (FDR < 0.05).

### Development of a LUAD hypoxia-associated signature

**Figure 1:**
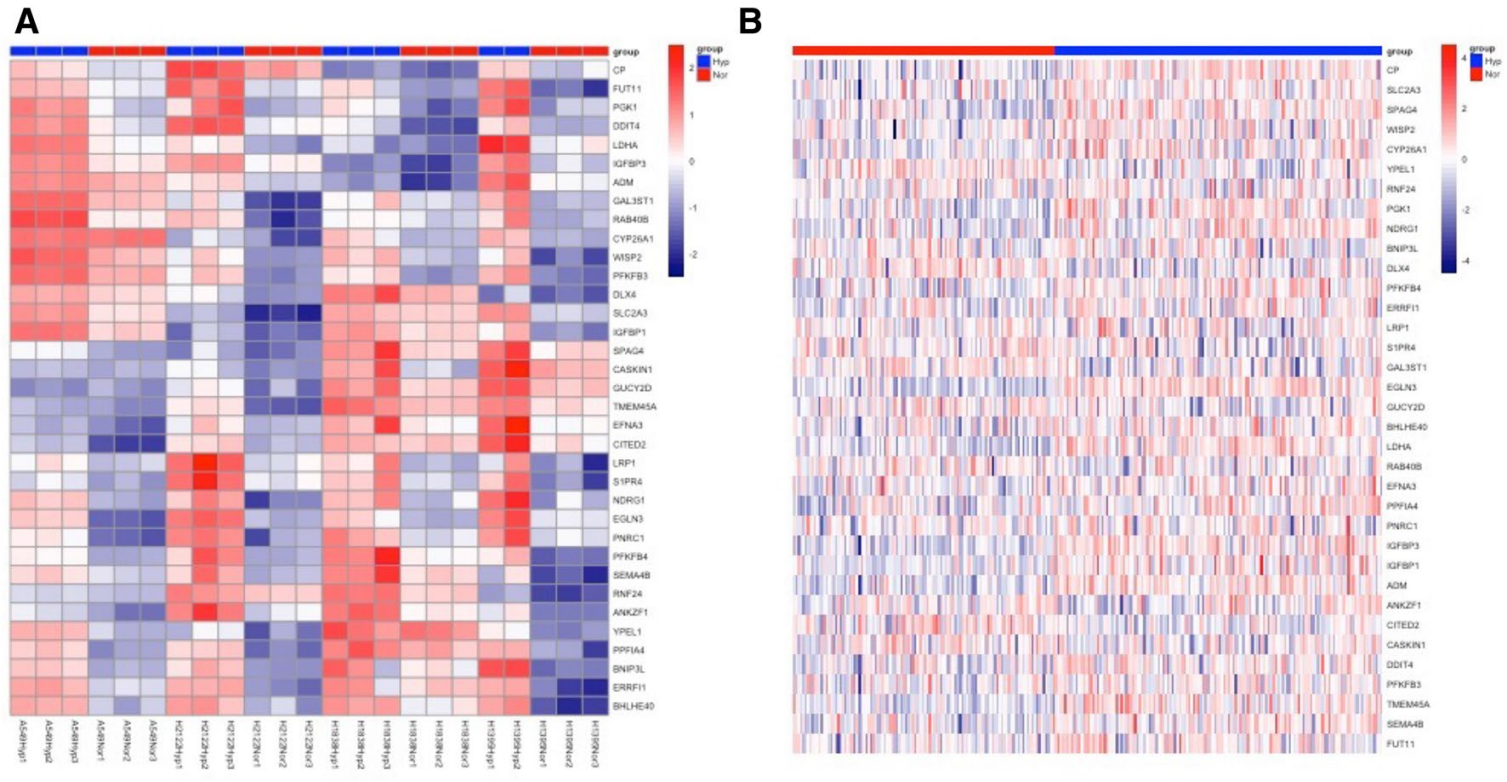
Genes clustered into hypoxia and normoxia phenotypes based on expression similarity of 35 seed genes used to develop the LUAD hypoxia-associated signature. Heatmaps are shown for four LUAD cell lines cultured in 1% O_2_ and 21% O_2_ (**A**) and 252 samples from the TCGA LUAD training dataset (**B**). *TCGA LUAD* The Cancer Genome Atlas lung adenocarcinoma.

To develop the LUAD signature, the 35 genes induced in ≥ 3 cell lines were used as seed genes (Fig. 1A; Supplementary Table 1). Enriched gene ontology and KEGG terms for the seed genes included processes involved in metabolism and response to hypoxia (Supplementary Tables 2 and 3). The Cancer Genome Atlas (TCGA)-LUAD cohort was split into training (n = 252) and test (n = 250) datasets. The seed genes were used to cluster the training dataset into two groups (n = 112, n = 140) based on their expression similarity (Fig. 1B). There were 1,198 genes significantly up-regulated (fold change > 1.5 and FDR < 0.05) in the 140 tumour group. Compared with the 112 group of the 35 seed genes: 15 were significantly up-regulated, 10 were up-regulated with a < 1.5 fold change, five had similar expression and five were significantly down-regulated (FDR < 0.05). Of those not in the seed gene list, the well-known hypoxia-associated *CA9* was the fifth most significantly up-regulated gene in the 140-tumour group. Gene set enrichment analysis identified 16 gene sets, which were highly enriched in the 140-tumour group, with hallmarks associated with hypoxia among the most enriched pathways (Supplementary Fig. 2). Gene enrichment analyses clearly showed that the two groups had distinct hypoxia phenotypes (hypoxia-high or hypoxia-low).

**Figure 2:**
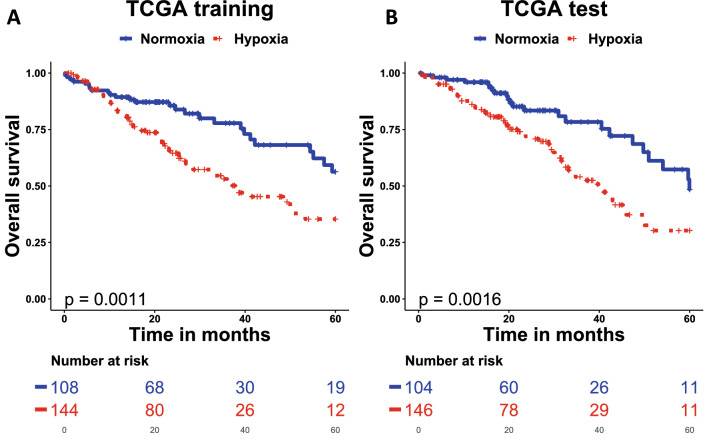
A 28-gene lung adenocarcinoma signature was prognostic for overall survival in TCGA LUAD. Patients with tumours stratified as hypoxic had worse overall survival in both TCGA LUAD training (**A**) and test (**B**) datasets. Patients were assigned to hypoxia or normoxia group based on the shrunken centroids. *TCGA LUAD* The Cancer Genome Atlas lung adenocarcinoma.

Prediction analysis for microarray (PAMR) selected 28 from the 35 seed genes, which gave the minimum tenfold cross validation error rate (Supplementary Table 4). PAMR assigned shrunken gene specific centroids to both groups with class labels hypoxia-high or hypoxia-low. The class label of a new sample was assigned based on closeness in square distance to the shrunken class centroid. The shrunken class centroids were computed using the shrunken gene specific centroids. The 28-gene signature was prognostic for overall survival in the TCGA LUAD training (n = 252; HR 2.12, 95% CI 1.34–3.37, p = 0.0011) and test (n = 250; HR 2.13, 95% CI 1.32–3.45, p = 0.0016) datasets (Fig. 2A, B, Table 1). The signature retained significance in a multivariable analysis in the training (HR 2.09, 95% CI 1.24–3.53, p = 0.0059) but not the test (HR 1.49, 95% CI 0.88–2.54, p = 0.14) dataset (Table 1).

**Table 1:** Univariable and multivariable analyses of the TCGA training and test datasets.

	Univariable		Multivariable	
	HR [95% CI]	p	HR [95% CI]	p
**TCGA LUAD training (n = 252)**				
Hypoxia	**2.12 [1.34–3.37]**	**0.0011**	**2.09 [1.24–3.53]**	**0.0059**
Stage (III&IV vs I&II)	**2.22 [1.40–3.50]**	**0.0005**	**1.93 [1.14–3.28]**	**0.015**
Male	1.03 [0.68–1.57]	0.90		
Age (continuous)	1.02 [1.00–1.04]	0.10		
Smoker (never)	1.02 [0.55–1.89]	1.00		
Surgical margin (R1&R2)	**3.91 [1.84–8.29]**	**0.0001**	**2.64 [1.20–5.84]**	**0.016**
**TCGA LUAD test (n = 250)**				
Hypoxia	**2.13 [1.32–3.45]**	**0.0016**	**1.49 [0.88–2.54]**	**0.14**
Stage (III&IV vs I&II)	**3.25 [2.08–5.09]**	**< 0.0001**	**3.04 [1.81–5.09]**	**< 0.0001**
Male	1.12 [0.73–1.72]	0.60		
Age (continuous)	0.99 [0.97–1.01]	0.40		
Smoker (never)	1.09 [0.64–1.85]	0.80		
Surgical margin (R1&R2)	3.80 [1.51–9.57]	0.002	2.69 [1.05–6.88]	0.038

Significant values are in bold.

### Validation of LUAD hypoxia signature

**Figure 3:**
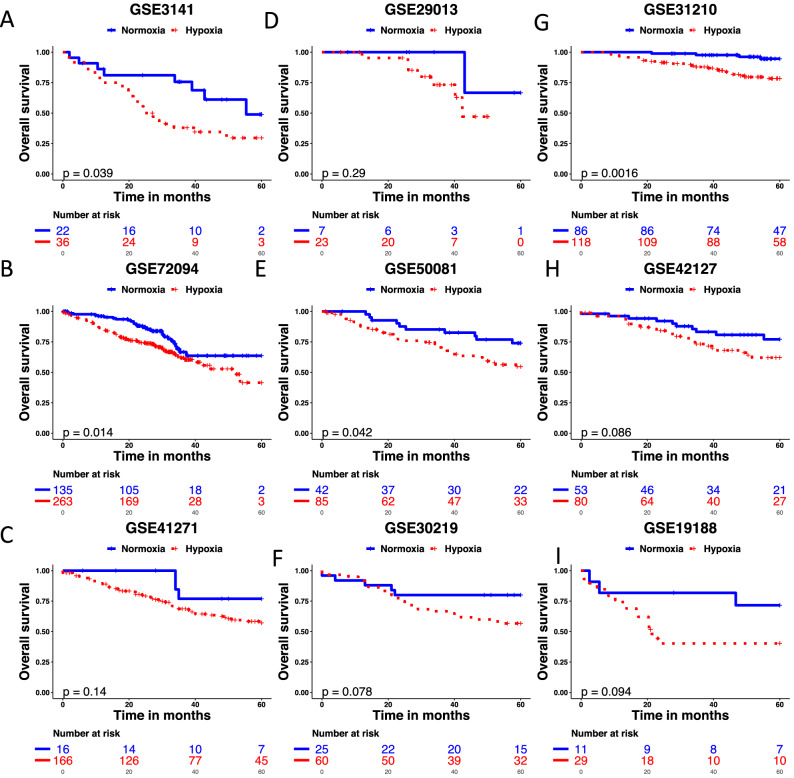
Perfomance of the 28-gene signature in nine independent cohorts. Kaplan–Meier curves for overall survival shown for nine LUAD validation datasets with tumours classified as hypoxic or normoxia based on centroids. *LUAD* lung adenocarcinoma.

**Figure 4:**
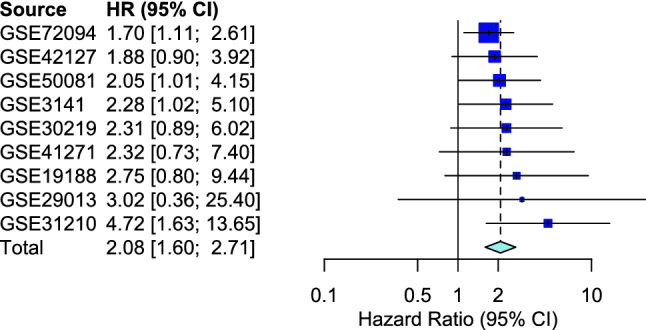
Meta analyses for the hazard ratios of the 28-gene LUAD hypoxia signature in nine expression datasets. A fixed effect model was used with the generic invariance method. *LUAD* lung adenocarcinoma.

The LUAD hypoxia signature was tested in nine independent cohorts profiled using different expression platforms (Fig. 3). The signature was prognostic for overall survival in four cohorts: GSE3141, GSE72094, GSE50081 and GSE31210 (p < 0.05). There was a trend towards prognostic significance for overall survival in three cohorts: GSE30219, GSE42127 and GSE19188 (p < 0.10). The signature had no prognostic significance for overall survival in two cohorts: GSE41271 and GSE29013 (p > 0.10). Multivariable analyses were carried out for cohorts where the signature was prognostic and clinico-pathological variables were available. The LUAD hypoxia signature retained prognostic significance for overall survival in GSE31210 (HR 3.72, 95% CI 1.26–11.01, p = 0.02) and GSE72094 (HR 1.57, 95% CI 1.02–2.41, p = 0.04), but lost prognostic significance in GSE50081 (HR 1.91, 95% CI 0.94–3.88, p = 0.07) (Table 2). In a univariable meta-analysis of the nine studies the signature was prognostic for overall survival (n = 1257; HR 2.08, 95% CI 1.60–2.70, p < 0.0001) (Fig. 4). A multivariable meta-analysis of four studies (TCGA-test, GSE31210, GSE72094 and GSE50091) showed the signature was an independent prognostic factor for overall survival (n = 979; HR 1.76, 95% CI 1.50–2.08, p < 0.0001).

**Table 2:** Univariable and multivariable analyses for LUAD validation datasets with p < 0.05 in univariate analyses for hypoxia and clinico-pathological variables available.

	Univariable		Multivariable	
	HR [95% CI]	p	HR [95% CI]	p
**GSE31210 (overall survival) (n = 204)**				
Hypoxia	**4.72 [1.63–13.7]**	**0.0016**	**3.72 [1.26–11.01]**	**0.02**
Stage (II vs I)	**4.07 [1.90–8.70]**	**0.00009**	**2.71 [1.23–5.96]**	**0.014**
Age (continuous)	1.02 [0.96–1.07]	0.60		
Male	**2.24 [1.02–4.88]**	**0.04**	**1.36 [0.50–3.68]**	**0.55**
Smoker (never)	**0.40 [0.18–0.88]**	**0.02**	**0.55 [0.20–1.54]**	**0.26**
**GSE72094 (overall survival) (n = 398)**				
Hypoxia	**1.70 [1.11–2.61]**	**0.014**	**1.57 [1.02–2.41]**	**0.04**
Stage (III&IV vs I&II)	**2.56 [1.72–3.82]**	**0.000002**	**2.78 [1.85–4.18]**	**0.0000009**
Age (continuous)	1.01 [0.99–1.03]	0.50		
Male	**1.55 [1.07–2.25]**	**0.02**	**1.74 [1.19–2.54]**	**0.004**
Smoker (never)	0.73 [0.32–1.68]	0.50		
**GSE50081 (overall survival) (n = 127)**				
Hypoxia	**2.05 [1.01–4.15]**	**0.042**	**1.91 [0.94–3.88]**	**0.07**
Stage (II vs I)	**2.19 [1.19–4.04]**	**0.009**	**2.06 [1.12–3.80]**	**0.02**
Age (continuous)	1.02 [0.99–1.06]	0.10		
Male	1.53[0.83–2.81]	0.20		
Smoker (never)	0.41 [0.14–1.13]	0.07		

Significant values are in bold.

The signature was tested for relapse-free survival in three cohorts (Supplementary Fig. 3). The signature was prognostic in GSE8894 in univariable analysis (multivariable was not possible due to lack of clinico-pathological data) and GSE31210 in both univariable (HR 3.31, 95% CI 1.73–6.54, p = 0.0001) and multivariable (HR 2.70, 95% CI 1.36–5.35, p = 0.004) analyses (Supplementary Table 5). The signature was not prognostic in GSE41271.

The performance of the LUAD signature was also examined in different cancers using gene expression data sets from TCGA. The signature was not prognostic in sarcoma (SARC, N = 259, HR 1.23, 95% CI 0.82–1.83, p = 0.32), prostate (PRAD, N = 497, HR 1.10, 95% CI 0.20–6.01, p = 0.92) or bladder (BLCA, N = 406, HR 0.85, 95% CI 0.63–1.14, p = 0.29) datasets. However, it was prognostic with the TCGA head and neck dataset (HNSC, N = 518, HR 1.42, 95% CI 1.05–1.90, p = 0.02).

### Association of the LUAD hypoxia signature with immune response

The hypoxia-high group was enriched in pathways involved in immune responses such as complement signalling pathway, interferon gamma response and inflammatory response (Supplementary Fig. 2). To investigate this further we used the ESTIMATE algorithm immune infiltration score^14^. There was a statistically significant higher ESTIMATE score in the hypoxia-high versus hypoxia-low groups in the TCGA-LUAD training (p = 0.044, Wilcoxon rank-sum test), but not the TCGA-LUAD test (p = 0.82, Wilcoxon rank-sum test) dataset. The CIBERSORT tool^15^ was then used to quantify 22 different types of immune cells (Supplementary Fig. 4). The hypoxia-high group had an increased infiltration of CD4 memory activated T cells, resting NK cells, M0 macrophages, M1 macrophages, regulatory T cells and activated mast cells. The hypoxia-high group had decreased infiltration of CD4 memory resting T cells, activated NK cells, monocytes and resting mast cells. In the TCGA-test dataset, the hypoxia-high group had an increased infiltration of CD4 memory activated T cells, resting NK cells, M0 macrophages, M1 macrophages and activated mast cells. There was decreased infiltration of CD4 memory resting T cells, resting dendritic cells, activated dendritic cells, monocytes and resting mast cells. In both the training and test datasets, the hypoxia high group had an increased infiltration of CD4 memory activated T cells, resting NK cells, M0 macrophages, M1 macrophages and activated mast cells.

### Comparison of the LUAD signature with other signatures

A comparison was made of the LUAD hypoxia signature’s performance with published hypoxia-associated signatures in the TCGA-LUAD training and test datasets (Table 3). Our 28-gene signature performed best in the TCGA-LUAD test dataset. The Winter and Buffa signatures also performed well in the TCGA training and test datasets^8,16^. Selected signatures were also trained on the TCGA training dataset using a method described previously^17^. Following training, only the 28-gene Lendhal and 15-gene Toustrup signatures were prognostic in the TCGA test cohort (Supplementary Table 6).

**Table 3:** Comparison of gene signatures in TCGA-LUAD train and test datasets.

	TCGA-LUAD train (p-value, HR [95% CI])	TCGA-LUAD test (p-value, HR [95% CI])
Winter (99-gene)	0.001, 2.05 [1.33–3.18]	0.003, 1.93 [1.24–3.01]
Buffa (51-gene)	0.0009, 2.08 [1.34–3.23]	0.003, 1.97 [1.25–3.08]
Chi (118-gene)	0.30, 1.26 [0.79–2.01]	0.07, 1.49 [0.97–2.31]
Lendhal (30-gene)	0.02, 1.63 [1.07–2.50]	0.30, 0.81 [0.52–1.25]
Toustrup (15-gene)	0.03, 1.60 [1.04–2.45]	0.08, 1.47 [0.96–2.27]
Sun (16-gene)	0.10, 1.38 [0.90–2.11]	0.05, 1.55 [1.00–2.39]
Shi (10-gene)	0.10, 1.42 [0.93–2.18]	0.20, 1.32 [0.86–2.03]
West (28-gene)	0.001, 2.12 [1.34–3.37]	0.0016, 2.13 [1.32–3.45]

We tested two of the three other developed hypoxia-associated LUAD signatures (Sun and Shi signatures) and the Buffa signature^8,10,11^ in the independent validation cohorts. The four-gene LUAD hypoxia-associated signature developed by Mo and colleagues could not be tested as the coefficients used in the signature were not constant^9^. The 16-gene Sun signature was not tested in GSE72094 as it was used to develop the signature. The Sun signature was only prognostic for overall survival in two (GSE50081, GSE31210) of the eight cohorts (GSE3141, GSE41271, GSE29013, GSE50081, GSE30219, GSE31210, GSE42127, GSE19188) with a trend towards prognostic significance in another two cohorts (GSE3141, GSE30219 (Supplementary Fig. 5). The Sun signature retained prognostic significance in GSE50081 (HR 2.41, 95% CI 1.29–4.50, p = 0.0058) but not GSE31210 (HR 2.14, 95% CI 0.86–5.34, p = 0.10) in multivariable analyses. In a univariable meta-analysis of the eight studies, the signature was prognostic for overall survival (n = 859; HR 1.81, 95% CI 1.41–2.34, p < 0.0001) (Supplementary Fig. 6).

The 10-gene Shi signature was tested in eight cohorts (GSE3141, GSE41271, GSE29013, GSE50081, GSE30219, GSE31210, GSE42127, GSE19188) (Supplementary Fig. 7). The Shi signature was not tested in GSE72094 as it was used to develop the signature. The Shi signature was prognostic in GSE50081 (HR 2.07, 95% CI 1.07–4.03, p = 0.028) (Supplementary Fig. 7D), and showed a trend towards prognostic significance in GSE31210 (HR 1.92, 95% CI 0.88–4.21, p = 0.095) and GSE42127 (HR 2.00, 95% CI 0.98–4.01, p = 0.051) (Supplementary Fig. 7F,G). In a univariable meta-analysis of the eight studies the signature was prognostic for overall survival (n = 859; HR 1.49, 95% CI 1.15–1.92, p < 0.0023) (Supplementary Fig. 8). In a multivariable analysis of GSE50081, the Shi signature retained prognostic significance (HR 1.98, 95% CI 1.02–3.84, p = 0.045).

The Buffa signature was tested in four cohorts (GSE72094, GSE41271, GSE4217, GSE3141) that had expression data for all 51 genes, and was prognostic in three (GSE72094, GSE41271, GSE4217) (Supplementary Fig. 9). In a univariable meta-analysis of the eight studies, the signature was prognostic for overall survival (n = 771; HR 1.92, 95% CI 1.48–2.49, p < 0.0001) (Supplementary Fig. 10). In a multivariable analysis, the signature retained prognostic significance in GSE72094 (HR 1.84, 95% CI 1.25–2.69, p = 0.0019). Supplementary Table 7 summarises the results of the univariable meta-analyses for four signatures. In terms of prognostication, our signature had similar performance to the Sun 16-gene.

### Failure to develop a lung squamous cell carcinoma hypoxia signature

The TCGA LUSC cohort was divided into training (n = 247) and test (n = 247) datasets. Our 28-gene LUAD signature was not prognostic in either the training (HR 0.97, 95% CI 0.56–1.66, p = 0.90) or test (HR 0.73, 95% CI 0.43–1.25, p = 0.20) datasets. We developed a signature using seed genes derived from RNAseq data generated for four LUSC cell lines (NCI-H520, NCI-H1703, NCI-H2170, NCI-H1869) (Supplementary Fig. 11). There were 500 genes up-regulated genes in hypoxia after removing cell line effects (“Methods”). Of the 500 genes, 494 were annotated in the TCGA LUSC dataset. The 494 genes were used to cluster the training dataset into two groups (n = 247 and n = 247). The cluster with significant enrichment for hypoxia (NES = 2.45, p = 0.0048) was assigned the hypoxia-high label. PAMR models of different sizes were created and a 23-gene signature derived, which was prognostic in the TCGA LUSC training (n = 247, HR 1.60, 95% CI 1.06–2.42, p = 0.03) and test (n = 247, HR 1.60, 95% CI 1.05–2.44, p = 0.03) datasets. However, the 23 gene signature failed to show prognostic significance in LUSC patients in GSE3141 (n = 53, HR 1.10, 95% CI 0.51–2.38, p = 0.80), GSE8894 (n = 75, HR 1.10, 95% CI 0.54–2.25, p = 0.79), GSE19188 (n = 24, HR 0.61, 95% CI 0.23–1.61, p = 0.33), GSE29013 (n = 25, HR 1.20, 95% CI 0.35–4.18, p = 0.77), GSE50081 (n = 42, HR 1.06, 95% CI 0.32–3.48, p = 0.92) and GSE42127 (n = 43, HR 0.56, 95% CI 0.21–1.51, p = 0.25). A meta-analysis of the external validation and TCGA-LUSC test datasets showed that the signature was not prognostic (n = 509, HR 1.15, 95% CI 0.87–1.52, p = 0.33). In addition, the expression data from the TCGA test data and external validation sets were pooled and scaled but still failed to achieve prognostic significance (n = 509, HR 1.08, 95% CI 0.81–1.44, p = 0.62).

Other signatures (Winter, Buffa, Chi. Lendhal, Toustrup) were also tested in the TCGA LUSC training and test datasets using their original methods, but none were prognostic (Supplementary Table 8).

## Discussion

A 28-gene LUAD hypoxia-associated signature was developed and validated in multiple independent cohorts. The signature was prognostic in gene expression datasets profiled using different approaches showing its transferability across platforms. Pathway analysis confirmed the association of the signature with hypoxia. We also derived a LUSC hypoxia-associated signature, which did not validate in multiple independent cohorts.

The 35 seed genes induced in at least three LUAD cell lines included common hypoxia induced genes such as *ADM*, *PGK1*, *NDRG1* and *BNIP3L*^6^. There were no downregulated genes in common between the LUAD cell lines in response to hypoxia. This finding reflects the heterogeneity in the response to hypoxia among the LUAD cell lines, which is also seen in the heatmap of the cell line data. There were no common genes between our LUAD hypoxia signature and the other three reported LUAD hypoxia signatures (Sun, Shi, Mo). The Buffa and our signature had four genes in common (*PGK1, NDRG1, DDIT4, ADM*), while the Mo and Buffa signatures had two genes in common (*SLC2A1, PFKP*). In terms of tumour type specificity, we used the same cell line approach to derive signatures for sarcoma^12^ and prostate cancer^13^, i.e., identified genes that changed expression in response to exposure to 1% oxygen for 24 h. The LUAD, prostate and sarcoma signatures had one gene in common (*BHLHE40*). The sarcoma and LUAD signatures had three common genes (*PPFIA4, BNIP3L, NDRG1*). The lack of similarity meant it was not possible to develop a signature based on overlapping genes across multiple models. The heterogeneity in hypoxia response reflects its roles in driving the phenotypic diversity of cancer cells in the tumour microenvironment that promotes metastasis and therapy resistance^18^.

Our 35 seed genes were used to cluster the TCGA-LUAD training dataset into two groups. One group was identified using gene enrichment analyses as enriched with hypoxia genes showing that our signature was associated with hypoxia. The hypoxia-high group was also enriched in pathways involved in the immune response such as complement signalling pathway, interferon gamma response and inflammatory response (Supplementary Fig. 2), which is consistent with the known cross-talk between hypoxia and inflammation^19^. The CIBERSORT scores showed that the more hypoxic tumours had increased infiltration of M0 macrophages, M1 macrophages, CD4 memory activated T cells and activated mast cells among other immune cells. Hypoxia associates with immune suppression and evasion in cancer^20,21^. It has been previously reported that hypoxia promotes M2 polarisation in hypoxia in LUAD^22^. However, the analyses show infiltration of M1 macrophages and CD4 memory activated T cells in the hypoxia high groups, which are both associated with immune activation^23,24^. These two immune cells were also increased in hypoxia high groups identified using another LUAD hypoxia signature^9^. Polarisation of macrophages towards an M1 phenotype has been associated with cycling hypoxia^25^ and the macrophage polarisation may be different dependent on the presence of cycling or chronic hypoxia.

The derived 28-gene LUAD hypoxia signature was prognostic in multiple independent validation datasets. There are likely to be several reasons why the signature was prognostic in most but not all the validation sets, e.g., inherent differences between the cohorts and the quality of data capture, differences in cohort sizes and methods used to generate gene expression data. Our signature outperformed most published signatures. One reason that the 28-gene LUAD hypoxia signature outperformed some signatures (Supplementary Figs. 5, 7) was that they were developed for early stage LUAD patients^10^. Our validation datasets included patients across all stages. We could not compare our signature with the four-gene LUAD hypoxia-associated signature developed for all stages^9^ as the coefficients in the signature were not constant. The Buffa signature performed well, but was not derived specifically for lung cancer. The Buffa signature is prognostic in multiple cancers^6,8^, but its predictive ability has not been tested in different cancers. Our group showed previously that the 26-gene West head and neck hypoxia signature predicted benefit from having hypoxia-modifying therapy with radiotherapy in head and neck but not bladder cancer patients^26^. The latter indicates the need for tissue-specific hypoxia-associated signatures. Our LUAD hypoxia signature was prognostic in a head and neck cancer dataset, but not in the other cancer types tested (sarcoma, prostate and bladder). However, based on our previous findings^26^ we expect a tumour-type specific signature will out-perform a common signature.

A limitation of our study was the inability to show prognostic significance for the LUAD hypoxia signature in all the cohorts. This may be due to the small size of some of the cohorts (GSE29013, GSE19188) and the signature assigning very few patients as having hypoxia low tumours (GSE41271) (Fig. 3). A second limitation of the study was the inability to validate a LUSC signature in multiple cohorts or in a combined validation cohort. LUSC is considered among the most hypoxic tumours^27^. It is possible that the seed genes from LUSC cell lines exposed to 0.2% O_2_ rather than 1% O_2_ would be needed to develop a LUSC hypoxia-associated signature. Another limitation was the inability to test whether our signatures predict benefit from having hypoxia-modifying treatments due to the lack of clinical trials in the area. A fourth limitation was the inability to test our signatures in patients undergoing radiotherapy where there is a high level of evidence that adding hypoxia-modifying treatments improve outcomes^28^. A fifth limitation was that our seed genes were derived using a single timepoint and oxygen concentration. We did not assess whether hypoxia-associated changes in cell lines are reversible or if measuring transcriptional changes at 24 h is relevant to long-term response to hypoxia. Due to the cost of RNA sequencing, it was not possible to quantify gene expression at multiple timepoints and oxygen levels or during reoxygenation. The latter would be worth exploring in a subsequent study to investigate whether another approach improved on signature performance. The use of 1% oxygen and 24 h exposure is common in the literature, but we know cellular responses to hypoxia involve multiple reversible and irreversible adaptive mechanisms^29^. It is noteworthy that Starmans et al.^30^ showed that only a subset of hypoxia-induced genes in vitro were prognostic in a clinical dataset and that, despite evidence of temporal patterns of gene-expression in vitro, the subset of prognostic hypoxia regulated genes could not be identified based on temporal pattern alone.

In conclusion, our 28-gene LUAD hypoxia signature validated in multiple cohorts with evidence of superiority of published signatures. Future work needs to validate the signature further using a gene expression platform suitable for routine clinical use and using RNA extracted from formalin-fixed, paraffin-embedded diagnostic biopsies. The signature can be tested for its predictive ability in a prospective setting in a clinical trial of radiotherapy with hypoxia-modifying treatment.

## Materials and methods

### Cell line work

LUAD (A549, NCI-H2122, NCI-H1395, NCI-H1838) and LUSC (NCI-H520, NCI-H1703, NCI-H2170, NCI-H1869) cell lines were purchased from the American Type Culture Collection (ATCC, Mannasas, USA). Cell lines were authenticated using the Promega Powerplex 21 system (Chilworth, UK) and regularly tested for mycoplasma (Molecular Biology Core Facility, Cancer Research UK Manchester Institute). All cell lines were grown in RPMI-1640 with l-glutamine (Thermo Fisher Scientific, Paisley, UK) supplemented with 10% foetal calf serum (FCS; Sigma-Aldrich, Dorset, UK/Lonza Biologics, Slough, UK) and penicillin–streptomycin (Sigma-Aldrich)**.**

The lung cancer cell lines were seeded at an appropriate density based on growth curves to obtain 60% confluency after 48 h incubation under normoxia (21% O_2_). After 24 h under normoxia (21% O_2_), media were changed and cells cultured in parallel in normoxia and hypoxia (1% O_2_). Hypoxia was obtained using the Ruskin Invivo2 400 hypoxia cabinet (Ruskin Technology Ltd, Bridgend, UK). After a further 24 h incubation, cells were harvested. RNA was then extracted using TRIzol-phenol chloroform (Thermo Fisher Scientific, Paisley, UK) followed by clean up using the RNAeasy mini kit (Qiagen) and removal of genomic DNA contamination using the Invitrogen™ DNA-*free*™ DNA Removal Kit (Thermo Fisher Scientific). Three independent experiments were carried out.

### Processing of lung cancer cell line RNA-seq data

Samples with RNA integrity number (RIN) ≥ 8 were sent for sequencing at the University of Manchester Genomic Technologies Core Facilities. Library prep was carried out using the Illumina TruSeq^®^ Stranded mRNA assay. Poly-A tail RNA-sequencing at 76 million reads paired-end was carried out in biological triplicate for the four lung cancer cell lines using the Illumina Hi-Seq4000. After sequence adapters were removed, reads were quality trimmed using Trimmomatic (v 0.36)^31^. The reads were mapped against the reference human genome (GRCH38). Using annotation from GENCODE version 27, counts per gene were calculated using STAR (v 2.5.3)^32^. Differential expression analysis was carried out using edgeR (v 3.26.8) with significantly expressed genes selected at FDR < 0.05. KEGG pathway analysis was carried out by kegga functions in edgeR, with terms classified as enriched at a non-adjusted p-value < 0.05.

### Development of the LUAD mRNA hypoxia-associated gene signature

Differential expression analysis was carried out with RNASeq data from four LUAD cell lines cultured in hypoxic and normoxic conditions. EdgeR (v 3.26.8) was used for expression analysis with scaled raw counts. Protein-coding genes induced under hypoxia in at least three LUAD cell lines (FDR < 0.05) were used as seed genes. TCGA-LUAD dataset was downloaded from the TCGA-LUAD RNA-Seq by Expectation Maximisation (RSEM) normalised counts had a pseudo-count of 1 added before being log_2_ transformed and median centred. TCGA-LUAD dataset was then split into a training (n = 252) and test (n = 250) dataset balanced on gender. The training dataset was clustered into two groups based on the expression similarity of the seed genes using k-means clustering. Differential expression analysis was carried out between the two k-means groups using LIMMA^33^. Gene set enrichment analysis was carried out using fgsea (v 1.10.1)^34^ on the up-regulated group of genes including seed genes. Gene set enrichment analysis confirmed that the two groups were associated with different hypoxia statuses and the hypoxic group was associated with hypoxia related processes. The GSEA enrichment association of the k-means groups were used to apply hypoxic or normoxic class labels to the tumour classification. The expression levels of seed genes in the hypoxic and normoxic group was also established in this way.

Using the hypoxia class labels, prediction analysis of microarray data (PAMR)^35^ was used to refine the signature genes, selecting the signature and shrinkage threshold that gave the minimum classification error rate after tenfold cross validation of the training test.

### Cohorts used for validation

The LUAD hypoxia signature was validated in ten cohorts for which expression and clinical data were downloaded using GEOquery from Gene Expression Omnibus (GEO)^36^. GSE3141, GSE31210, GSE19188, GSE29013, GSE50081, GSE30219 and GSE8894 were profiled using Affymetrix Human Genome U133 plus 2.0 arrays. GSE41271 and GSE42127 was profiled using the Illumina HumanWG-6 v 3.0 expression beadchip and GSE72094 using the Rosetta/Merck Human RSTA Affymetrix 2.0 microarray. When multiple probesets mapped to a single gene, outlier probesets were removed and among the remaining probesets, the one with the highest median absolute deviation was selected.

### ESTIMATE and CIBERSORT analyses to study immune infiltration

ESTIMATE^14^ immune infiltration scores were downloaded from (https://bioinformatics.mdanderson.org/public-software/estimate). Non log-2 transformed FPKM (fragments per kilobase of exon per million mapped reads) RNA-seq data for the TCGA-LUAD training and test datasets were uploaded in the CIBERSORT^15^ tool (https://cibersort.stanford.edu/runcibersort.php). FPKM values were used instead of RSEM values as they have been tested to be superior in linearity space^37^. CIBERSORT was run using the default LM22 signature gene set, relative and absolute modes together, 100 permutations and quantile normalisation disabled as recommended for RNA-seq data. CIBERSORT absolute immune fraction scores for 22 immune cell populations for the datasets was the selected output from the CIBERSORT tool. For the analyses, only patients with statistically significant deconvolution results across all subsets were used. Wilcoxon rank-sum tests were calculated for each immune population between the hypoxia-high and hypoxia-low groups in each dataset (TCGA-LUAD training and test). GraphPad Prism (San Diego, CA, USA) version 9.0.0 (www.graphpad.com) was used to visualise the violin plots for the immune populations.

### Comparison of the LUAD hypoxia signature with other signatures

A comparison of the derived LUAD hypoxia signatures was made with seven other signatures (Winter, Buffa, Chi, Lendhal, Toustrup, Sun and Shi) in the TCGA training and test datasets according to their published methods^8,10,16,38–40^. The signatures were also compared with the LUAD hypoxia signatures in the TCGA test dataset after training on the TCGA training dataset using a previously described method^17^. The Buffa, Sun and Shi signature were also studied in the independent validation datasets using their published methods.

### Development of the lung squamous cell carcinoma hypoxia signature

Differential expression analysis was carried out using edgeR with RNASeq data from four LUSC cell lines cultured in hypoxic and normoxic conditions using only oxygen concentration as a factor. Cell lines specific batch effects were removed using ComBat from the sva package^41^. TCGA lung squamous cell carcinoma dataset was split into equal sized (n = 247) training and test datasets. Differentially expressed genes were used as seeds genes to build PAMR models of different sizes in an internal cross validation on prognosis using the TCGA lung squamous cell carcinoma training and test datasets. A 23 gene model that was prognostic in both training and test sets during internal validation was chosen for independent validation. The selected PAMR model with no shrinkage was used for validation in LUSC patients from GSE3394, GSE8894, GSE19188, GSE29013, GSE50081 and GSE42127 datasets.

### Statistical analysis

Data analyses were carried out using R version 3.6.1 (R core team, Vienna, Austria).

The Cox regression model was used for univariable and multivariable analyses of datasets. Log-rank test and Wald statistic compared differences in univariable and multivariable analyses, respectively. Variables with *p* value < 0.05 were used in the multivariable analysis models. Clinical end points were overall survival and relapse free survival. Data were censored at 5 years. The R package survival (v 3.1-12) was used for Cox analyses. The R package survminer (v 0.4.6) was used to plot Kaplan–Meier curves. Meta analyses was carried out using the meta package^42^ using the generic inverse variance method in a fixed effect model.

## Supplementary Information

Supplementary Information.

## Data Availability

All data were publicly available from TCGA and GEO datasets.
