# Supplementary material for: Development and validation of a hypoxia-associated signature for lung adenocarcinoma
Source: Sci Rep. 2022 Jan 25;12:1290. doi: 10.1038/s41598-022-05385-7 (PMC8789914; doi:10.1038/s41598-022-05385-7)
Supplement: Supplementary file 1 — Supplementary Information. [file 41598_2022_5385_MOESM1_ESM.docx]

Supplementary Information

Development and validation of a hypoxia-associated signature for lung adenocarcinoma

Brian Lane^†1^, Mairah T. Khan^†1^, Ananya Choudhury^1^, Ahmed Salem^2^, Catharine M.L West*^1^

^†^These two authors contributed equally to the work

Supplementary Table 1. Thirty five seed genes used to derive the lung adenocarcinoma hypoxia signature

| Genes | Name |
| --- | --- |
| CP | ceruloplasmin (ferroxidase) |
| SLC2A3 | solute carrier family 2 (facilitated glucose transporter), member 3 |
| SPAG4 | sperm associated antigen 4 |
| WISP2 | WNT1 inducible signaling pathway protein 2 |
| CYP26A1 | cytochrome P450, family 26, subfamily A, polypeptide 1 |
| YPEL1 | yippee like 1 |
| RNF24 | ring finger protein 24 |
| PGK1 | phosphoglycerate kinase 1 |
| NDRG1 | N-myc downstream regulated 1 |
| BNIP3L | BCL2/adenovirus E1B 19kDa interacting protein 3-like |
| DLX4 | distal-less homeobox 4 |
| PFKFB4 | 6-phosphofructo-2-kinase/fructose-2,6-biphosphatase 4 |
| ERRFI1 | ERBB receptor feedback inhibitor 1 |
| LRP1 | LDL receptor related protein 1 |
| S1PR4 | sphingosine-1-phosphate receptor 4 |
| GAL3ST1 | galactose-3-O-sulfotransferase 1 |
| EGLN3 | egl-9 family hypoxia-inducible factor 3 |
| GUCY2D | guanylate cyclase 2D, membrane (retina-specific) |
| BHLHE40 | basic helix-loop-helix family, member e40 |
| LDHA | lactate dehydrogenase A |
| RAB40B | RAB40B, member RAS oncogene family |
| EFNA3 | ephrin-A3 |
| PPFIA4 | protein tyrosine phosphatase, receptor type, f polypeptide (PTPRF), interacting protein (liprin), alpha 4 |
| PNRC1 | proline-rich nuclear receptor coactivator 1 |
| IGFBP3 | insulin like growth factor binding protein 3 |
| IGFBP1 | insulin like growth factor binding protein 1 |
| ADM | adrenomedullin |
| ANKZF1 | ankyrin repeat and zinc finger domain containing 1 |
| CITED2 | Cbp/p300-interacting transactivator, with Glu/Asp rich carboxy-terminal domain, 2 |
| CASKIN1 | CASK interacting protein 1 |
| DDIT4 | DNA damage inducible transcript 4 |
| PFKFB3 | 6-phosphofructo-2-kinase/fructose-2,6-biphosphatase 3 |
| TMEM45A | transmembrane protein 45A |
| SEMA4B | sema domain, immunoglobulin domain (Ig), transmembrane domain (TM) and short cytoplasmic domain, (semaphorin) 4B |
| FUT11 | fucosyltransferase 11 (alpha (1,3) fucosyltransferase) |

Supplementary Table 2. Gene Ontology pathway analyses of the thirty five lung adenocarcinoma seed genes

| Gene Ontology pathways | p-value |
| --- | --- |
| response to hypoxia | 9.30E-08 |
| response to decreased oxygen levels | 1.20E-07 |
| response to oxygen levels | 1.97E-07 |
| glycolytic process | 1.19E-06 |
| ATP generation from ADP | 1.24E-06 |
| ADP metabolic process | 1.73E-06 |
| nucleoside diphosphate phosphorylation | 2.44E-06 |
| nucleotide phosphorylation | 2.62E-06 |
| response to abiotic stimulus | 2.70E-06 |
| purine nucleoside diphosphate metabolic process | 2.72E-06 |
| purine ribonucleoside diphosphate metabolic process | 2.72E-06 |
| ribonucleoside diphosphate metabolic process | 3.03E-06 |
| pyruvate metabolic process | 4.69E-06 |
| nucleoside diphosphate metabolic process | 5.16E-06 |
| 6-phosphofructo-2-kinase activity | 1.57E-05 |
| carbohydrate catabolic process | 1.78E-05 |
| cellular response to hypoxia | 2.15E-05 |
| fructose 2,6-bisphosphate metabolic process | 2.61E-05 |
| fructose-2,6-bisphosphate 2-phosphatase activity | 2.61E-05 |
| cellular response to decreased oxygen levels | 2.69E-05 |

Supplementary Table 3. KEGG pathway analyses of the thirty five lung adenocarcinoma seed genes

| KEGG Pathway | p-value |
| --- | --- |
| HIF-1 signaling pathway | 0.00011 |
| Fructose and mannose metabolism | 0.0027 |
| Glycolysis / Gluconeogenesis | 0.011 |
| Mitophagy - animal | 0.011 |
| AMPK signaling pathway | 0.032 |

Supplementary Table 4. Twenty eight genes in the lung adenocarcinoma hypoxia signature

| Genes | Name |
| --- | --- |
| PFKFB4 | 6-phosphofructo-2-kinase/fructose-2,6-biphosphatase 4 |
| GUCY2D | Guanylate cyclase 2D, membrane (retina-specific) |
| CP | Ceruloplasmin (ferroxidase) |
| RNF24 | Ring finger protein 24 |
| TMEM45A | Transmembrane protein 45A |
| EGLN3 | Egl-9 family hypoxia-inducible factor 3 |
| PFKFB3 | 6-phosphofructo-2-kinase/fructose-2,6-biphosphatase 3 |
| BHLHE40 | Basic helix-loop-helix family, member e40 |
| PGK1 | Phosphoglycerate kinase 1 |
| RAB40B | RAB40B, member RAS oncogene family |
| PNRC1 | Proline-rich nuclear receptor coactivator 1 |
| PPFIA4 | Protein tyrosine phosphatase, receptor type, f polypeptide (PTPRF), interacting protein (liprin), alpha 4 |
| S1PR4 | Sphingosine-1-phosphate receptor 4 |
| BNIP3L | BCL-2/adenovirus E1B 19kDa interacting protein 3-like |
| NDRG1 | N-myc downstream regulated 1 |
| IGFBP1 | Insulin like growth factor binding protein 1 |
| YPEL1 | Yippee like 1 |
| FUT11 | Fucosyltransferase 11 (alpha (1,3) fucosyltransferase) |
| CASKIN1 | CASK interacting protein 1 |
| DDIT4 | DNA damage inducible transcript 4 |
| GAL3ST1 | Galactose-3-O-sulfotransferase 1 |
| SPAG4 | Sperm associated antigen 4 |
| DLX4 | Distal-less homeobox 4 |
| ERRFI1 | ERBB receptor feedback inhibitor 1 |
| SEMA4B | Sema domain, immunoglobulin domain (Ig), transmembrane domain (TM) and short cytoplasmic domain, (semaphorin) 4B |
| LRP1 | LDL receptor related protein 1 |
| ADM | Adrenomedullin |
| CYP26A1 | Cytochrome P450, family 26, subfamily A, polypeptide 1 |

Supplementary Table 5. Univariate and multivariate analyses for relapse free survival of GSE31210

| GSE31210 (Relapse free survival) | Univariate | | Multivariate | |
| --- | --- | --- | --- | --- |
|  | HR [95% CI] | p | HR [95% CI] | p |
| Hypoxia | 3.37 [1.73-6.54] | 0.0001 | 2.70 [1.36-5.35] | 0.004 |
| Stage (II vs I) | 3.55 [2.03-6.21] | 0.000002 | 2.75 [1.36-5.35] | 0.0006 |
| Male | 1.46 [0.85-2.50] | 0.20 |  |  |
| Age (continuous) | 1.03 [0.99-1.07] | 0.10 |  |  |
| Smoker (Never) | 0.67 [0.39-1.16] | 0.20 |  |  |

Supplementary Table 6. Comparison of the gene signatures in TCGA test dataset after model building using the TCGA training dataset

|  | TCGA test  (p-value, HR [95%CI]) |
| --- | --- |
| Winter | 0.08, 1.47 [0.95-2.27] |
| Buffa | 0.03, 1.62 [1.05-2.52] |
| Chi | 0.08, 1.48 [0.96-2.28] |
| Lendhal | 0.005,1.86 [1.20-2.90] |
| Toustrup | 0.006,1.84 [1.18-2.86] |

Supplementary Table 7. Comparison of the performance of different gene signatures in a meta-analysis of LUAD datasets

| Signature | n | HR | 95% CI | p-value |
| --- | --- | --- | --- | --- |
| 28 gene | 1257 | 2.08 | 1.60-2.70 | <0.0001 |
| Sun 16-gene | 859 | 1.81 | 1.41-2.34 | <0.0001 |
| Shi 10-gene | 859 | 1.49 | 1.15-1.92 | 0.0023 |
| Buffa 51-gene | 771 | 1.92 | 1.25-2.69 | .0019 |

Supplementary Table 8. Performance of the different gene signatures in TCGA lung squamous cell carcinoma train and test datasets

|  | TCGA train  (p-value, HR [95%CI]) | TCGA test  (p-value, HR [95%CI]) |
| --- | --- | --- |
| Winter (99-gene) | 0.70, 1.09 [0.72-1.64] | 0.60, 1.11 [0.73-1.68] |
| Buffa (51-gene) | 0.50, 1.16 [0.77-1.74] | 0.30, 1.27 [0.84-1.93] |
| Chi (118-gene) | 0.06, 0.66 [0.42-1.03] | 0.20, 0.75 [0.49-1.15] |
| Lendhal (30-gene) | 0.40, 0.83 [0.54-1.27] | 0.40, 1.21 [0.80-1.83] |
| Toustrup (15-gene) | 0.40, 1.21 [0.80-1.82] | 0.80, 1.06 [0.70-1.61] |

Supplementary Figure 1. MDS plots for hypoxia and normoxia repeats used in the LUAD analyses.

MDS: multidimensional scaling; LUAD: lung adenocarcinoma.


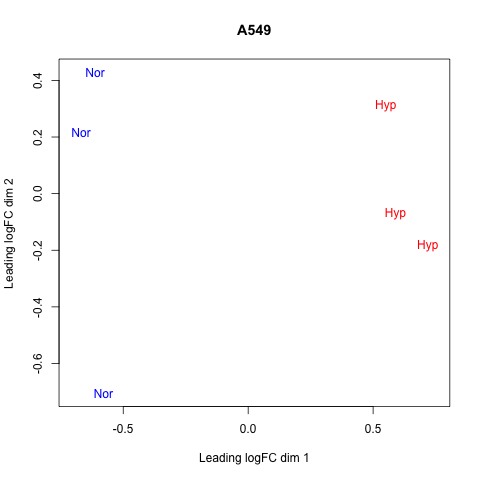

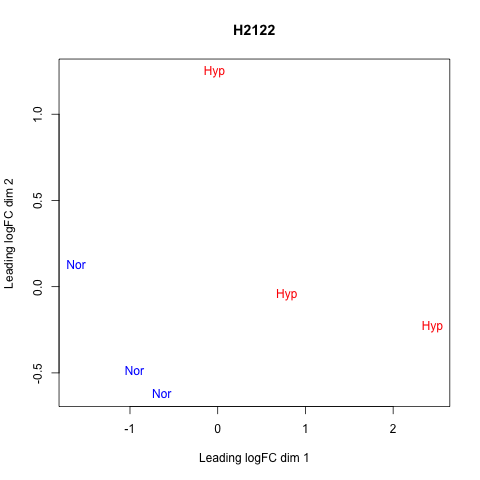

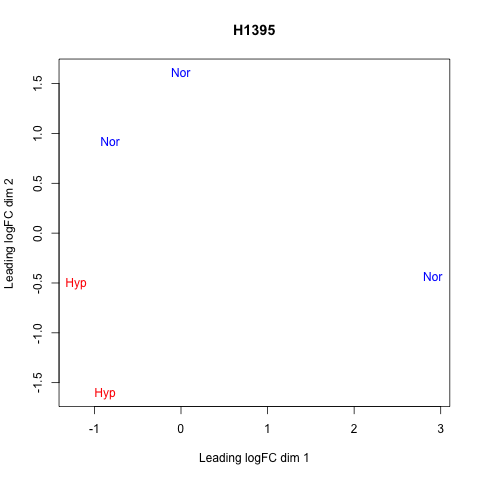

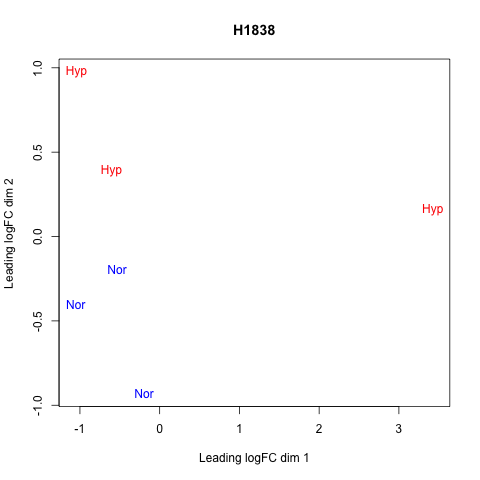


Supplementary Figure 2. Hypoxia is the most enriched process in the GSEA analyses.

GSEA was carried out on differentially expressed genes between the hypoxic and normoxic groups identified by k-means clustering in the TCGA training dataset.

GSEA: gene set enrichment analyses

Supplementary Figure 3. Kaplan Meier curves for relapse free survival for the hypoxia gene signature in LUAD validation datasets.

LUAD: lung adenocarcinoma

A

B

C

Supplementary Figure 4. Violin plots for the difference in CIBERSORT absolute immune fraction score between high-hypoxia and low-hypoxia groups in TCGA training (A) and test datasets (B).

Red represents the high-hypoxia group and blue represents the low hypoxia group.

* p-value<0.05, ** p-value<0.01, *** p-value<0.001, where p-value of Wilcoxin rank-sum test.

B

A

Supplementary Figure 5. Kaplan Meier curves for LUAD validation datasets by the 16 gene Sun signature.

Stratification by the median of the signature score

A

G

D

B

C

F

E

H

Supplementary Figure 6. Meta analyses for the hazard ratios of the Sun signature in eight expression datasets. A fixed effect model was used with the generic invariance method.

Supplementary Figure 7. Kaplan Meier curves for LUAD validation datasets by the ten gene Shi signature.

Stratification based on normalized score value of zero.

A

D

G

C

B

F

E

H

Supplementary Figure 8. Meta analyses for the hazard ratios of the Shi signature in eight expression datasets. A fixed effect model was used with the generic invariance method.

Supplementary Figure 9. Kaplan Meier curves for LUAD validation datasets by the 51 gene Buffa signature.

Stratification by the median of the signature score

B

A

D

C

Supplementary Figure 10. Meta analyses for the hazard ratios of the Buffa signature in four expression datasets. A fixed effect model was used with the generic invariance method.

Supplementary Figure 11. MDS plots for hypoxia and normoxia repeats used in the lung squamous cell carcinoma analyses.

One hypoxic repeat was removed from H1703 as it was an outlier

MDS: multidimensional scaling


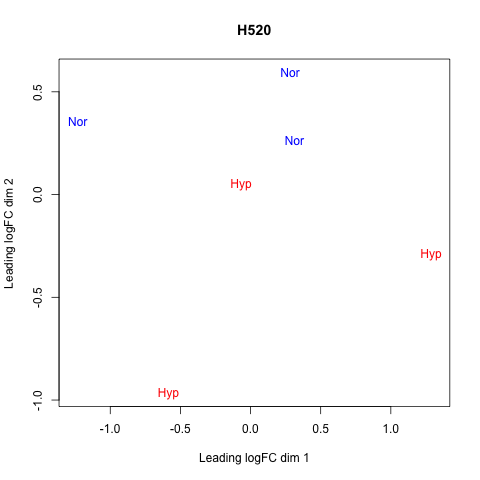

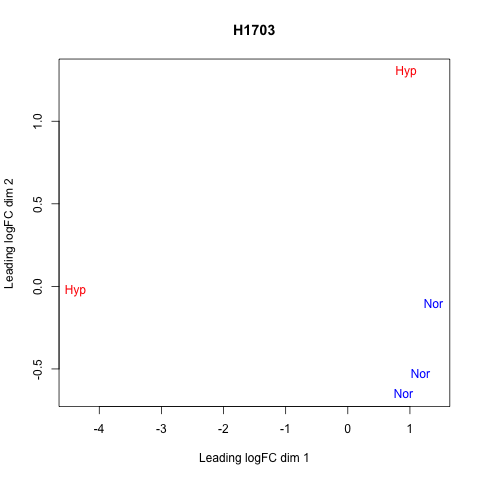

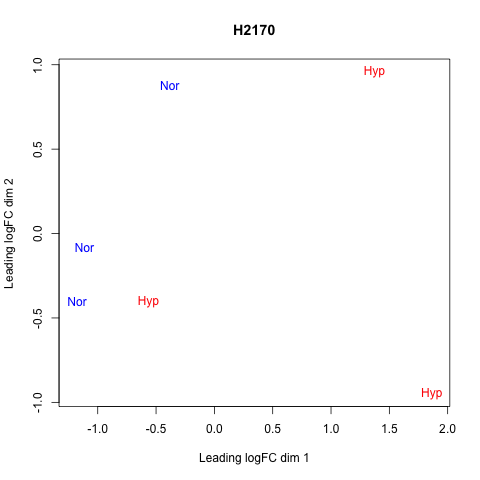

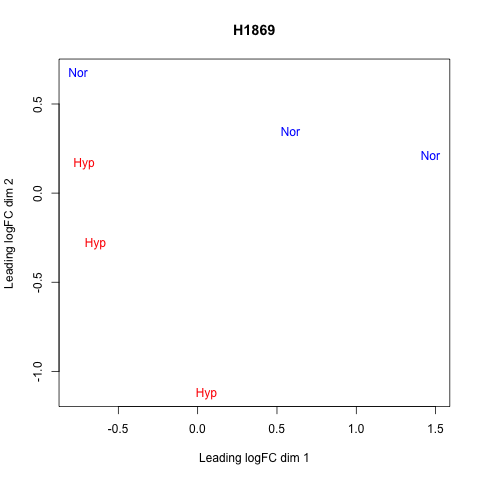


A

B

C

D
